# Effect of quadratus lumborum block on postoperative cognitive function in elderly patients undergoing laparoscopic radical gastrectomy: a randomized controlled trial

**DOI:** 10.1186/s12877-021-02179-w

**Published:** 2021-04-09

**Authors:** Manhua Zhu, Yong Qi, Huijuan He, Song Zhang, Yuliu Mei

**Affiliations:** 1 Department of Anesthesiology, Ningbo Medical Center Lihuili Hospital, Ningbo University, No.1111 Jiangnan Road, Zhejiang 315040 Ningbo, China; 2 Department of Anesthesiology,Ningbo Beilun People’s Hospital, No1288 Lushan East Road, Zhejiang 315800 Ningbo, China

**Keywords:** Quadratus lumborum block, Laparoscopic radical gastrectomy, Postoperative cognitive dysfunction, Elderly

## Abstract

**Background:**

Quadratus lumborum block (QLB) is a novel and effective postoperative analgesia method for abdominal surgeries. However, whether QLB can affect early postoperative cognitive function by inhibiting surgical traumatic stress and the inflammatory response remains unclear. This study aimed to explore the effect of QLB on postoperative cognitive function in elderly patients undergoing laparoscopic radical gastrectomy.

**Methods:**

Sixty-four elderly patients who underwent laparoscopic radical gastrectomy were randomly divided into the QLB group (Q group, *n* = 32) and control group (C group, *n* = 32). The Mini-Mental State Examination (MMSE) and Montreal Cognitive Assessment (MoCA) were used to measure cognitive function 1 day before and 7 days after surgery. Postoperative cognitive dysfunction (POCD) was defined as a decline of ≥ 1 SD in both tests. The visual analog scale (VAS) scores 6 h (T1), 24 h (T2), and 48 h (T3) after surgery were measured. The serum levels of high mobility group box protein 1 (HMGB1), interleukin-6 (IL-6), and tumor necrosis factor-α (TNF-α) were evaluated 1 day before surgery (baseline), and 1 day (day 1) and 3 days after surgery (day 3). The intraoperative remifentanil dosage, sufentanil consumption 24 h after surgery, recovery time from anesthesia, and adverse effects were also compared.

**Results:**

POCD was present in two patients in the QLB group and eight patients in the C group 7 days after surgery (6.7 % vs. 27.6 %, *P* = 0.032). The MMSE and MoCA scores were similar in both groups preoperatively, and the two scores were higher in the QLB group than in the C group 7 days after surgery (*P* < 0.05). The VAS scores were significantly lower in the Q group at all times after surgery (*P* < 0.05). Compared with the C group, the levels of HMGB1, TNF-α, and IL-6 were significantly decreased 1 and 3 days after surgery in the QLB group (*P* < 0.05). The remifentanil consumption intraoperatively and sufentanil 24 h postoperatively were significantly lower in the QLB group (*P* < 0.05). The recovery time from anesthesia was shorter in the QLB group (*P* < 0.05). No severe adverse effects occurred in either group.

**Conclusions:**

QLB could improve postoperative cognitive function in elderly patients undergoing laparoscopic radical gastrectomy. This may be related to the suppression of the inflammatory response after surgery.

**Trial registration:**

Chictr.org.cn identifier ChiCTR1900027574 (Date of registry: 19/11/2019, prospectively registered).

## Background

Due to the growing elderly population worldwide, an increasing number of elderly patients require surgery. Postoperative cognitive dysfunction (POCD) is a frequent complication among senile patients after anesthesia, with an incidence of 54.3 % at 6 weeks and 46.1 % at 1 year after surgery [1]. The majority of patients with gastric cancer are elderly, and gastrectomy is the primary treatment for this disease. Despite the advantages of a small incision, laparoscopic radical gastrectomy can also lead to persistent postoperative pain, which may increase the incidence of postoperative complications, such as POCD, and seriously affect patient prognosis and quality of life. Many risk factors, such as insufficient postoperative analgesia, limited mobility after surgery, improper use of drugs, the release of inflammatory mediators, and stress response, can contribute to the development of POCD [2]. Surgery leads to a systemic inflammatory response, and inflammatory mediators, such as interleukin-6 (IL-6), tumor necrosis factor-α (TNF-α), and interleukin-1β (IL-1β), are released into the plasma, resulting in POCD [3].

High mobility group box protein 1 (HMGB1) is an important proinflammatory cytokine. Studies have reported that HMGB1 can mediate systemic inflammatory responses and lead to neuroinflammation and cognitive decline [4]. It was reported that HMGB1 is correlated with cognitive impairment after gastrointestinal surgery [5].

Based on the strategy of enhanced recovery after surgery, multimodal analgesia after surgery is recommended to reduce the use of opioids, accelerate gastrointestinal function recovery, and minimize perioperative complications [6]. As an emerging regional block, quadratus lumborum block (QLB), which was first described by Blanco [7], was shown to provide somatic and visceral analgesia in abdominal wall and hip surgery, with the block dermatomes at T6-L1 [8] and improve the prognosis of patients. Sufficient postoperative analgesia can decrease stress and inflammatory reactions caused by surgery [9]. However, whether QLB can alleviate POCD by inhibiting the expression of serum HMGB1 remains unknown. In light of the above, we designed this prospective randomized controlled trial to examine the hypothesis that QLB is associated with a reduced occurrence of POCD in elderly patients undergoing laparoscopic radical gastrectomy. We further measured whether postoperative serum concentrations of HMGB1, TNF-α and IL-6 changed after QLB, as they are crucial inflammatory cytokines related to POCD.

## Methods

**Fig. 1:**
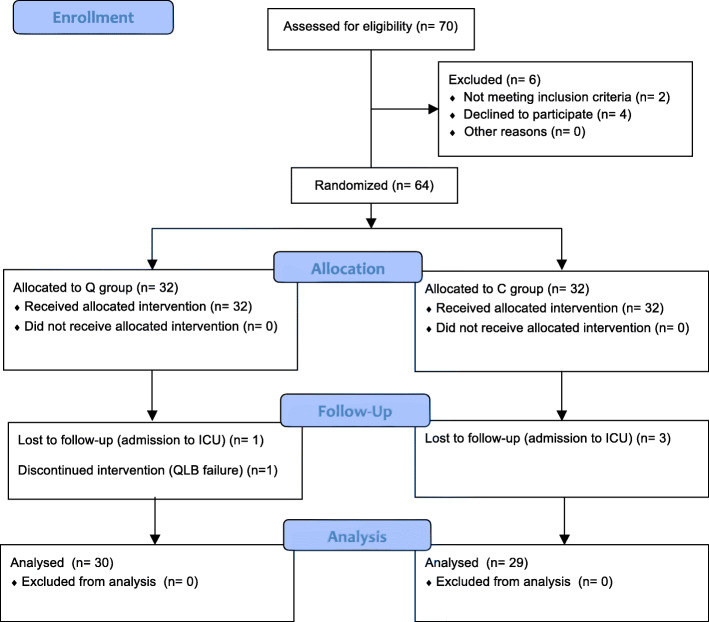
Flow diagram of the participants

The study was approved by the Ethics Committee of Ningbo Medical Center Lihuili Hospital, China (KY2019PJ018) and was registered with the Chinese Clinical Trial Registry (ChiCTR1900027574) before the enrollment process. After obtaining written informed consent, 70 patients undergoing laparoscopic radical gastrectomy from January 2020 to May 2020 in Ningbo Medical Center Lihuili Hospital were enrolled. All patients were randomly divided into the QLB group (Q group) and control group (C group) (Fig. 1). The inclusion criteria were as follows: ASA I-III; age > 65 years; received elementary education or above; Mini-Mental State Examination (MMSE) score ≥ 24 one day before surgery; and body mass index (BMI) < 31 kg/m^2^. The exclusion criteria included serious diseases of the cardio-cerebral vascular or respiratory system, hepatorenal dysfunction, history of mental or neurological disease, long-term use of antipsychotic or narcotic analgesics, history of alcohol abuse, severe speaking, hearing or vision impairment, and allergies to local anesthetics.

### Study protocol

**Fig. 2:**
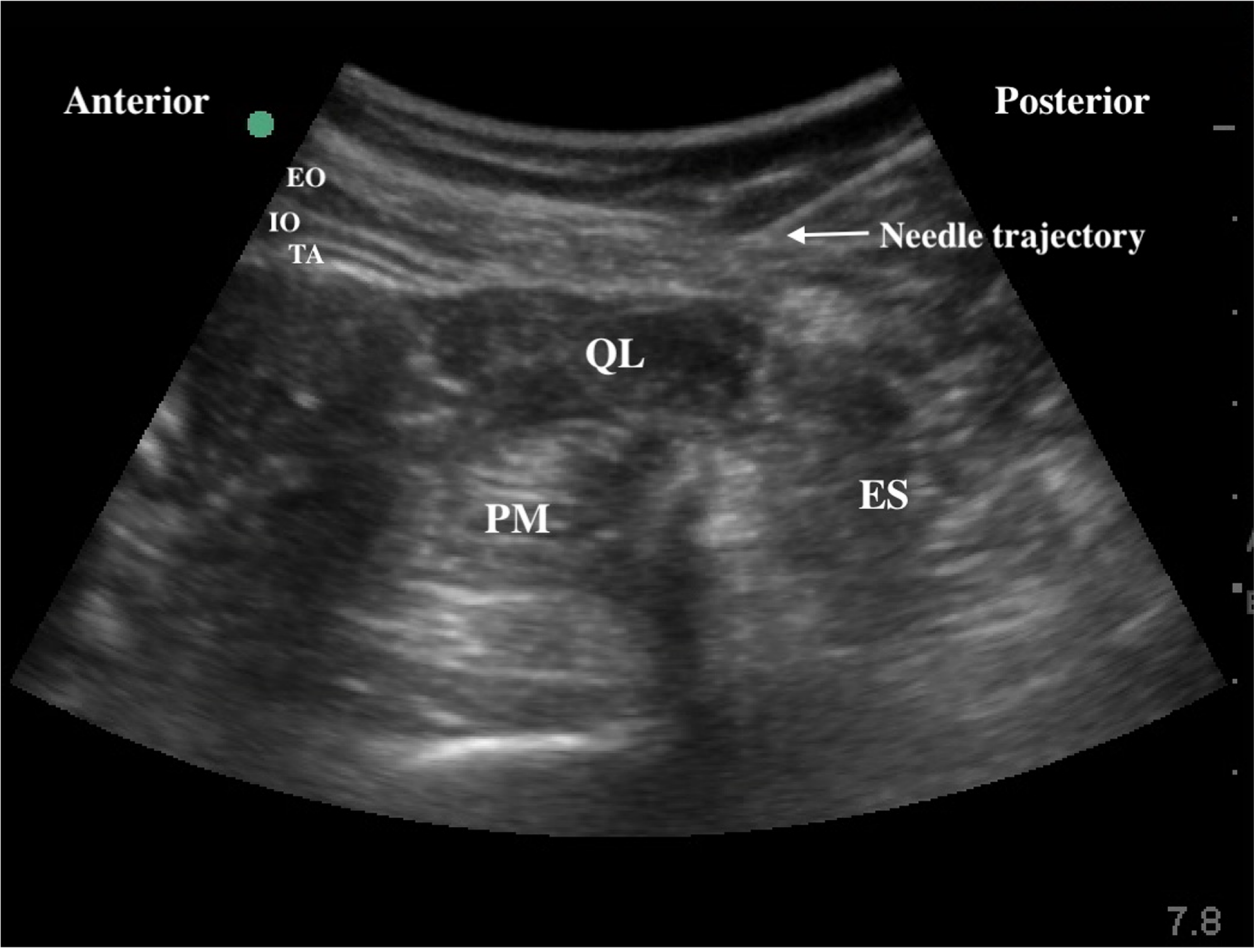
Ultrasonographic image of QLB. Arrow shows the direction of needle. EO, external oblique muscle; IO, internal oblique muscle; TA, transversus abdominis; QL, quadratus lumborum; PM, psoas major; ES, erector spinae

Invasive radial arterial blood pressure, heart rate (HR), electrocardiogram (ECG), percutaneous oxygen saturation (SpO_2_), and bispectral index (BIS) were routinely monitored after the patients entered to the operating room. Peripheral intravenous access and right central vein access were established. Prior to anesthesia induction, patients in both groups received ultrasound-guided bilateral QLB in the lateral position. Following disinfection of the intervention area, a convex probe (2–5 HZ, Edge, Sonosite, Seattle, USA) was positioned on the anterosuperior iliac crest and moved cranially until the external oblique, internal oblique, and transversus abdominis were identified. Then, the probe was moved posteriorly, and the quadratus lumborum muscle was observed. A 22 gauge × 80 mm needle (Kindly, Shanghai, China) was inserted into the posterior part of the QL muscle. After confirming the optimal site by hydrodissection, group Q was injected with 20 mL of 0.3 % ropivacaine (Naropin, AstraZeneca AB Company, Södertälje, Sweden), and group C was injected with 20 mL of 0.9 % saline on each side. Posterior spread was observed (Fig. 2). After the block, the dermatomes of the sensory block at the 15th minutes were evaluated using pinprick for all subjects.

After QLB, 0.02–0.03 mg/kg midazolam, 0.3–0.4 µg/kg sufentanil, 1.5–2 mg/kg propofol, and 0.6–0.8 mg/kg rocuronium were administered intravenously for anesthesia induction. Mechanical ventilation was performed by volume-controlled ventilation after intubation to maintain the end-tidal carbon dioxide pressure (PetCO_2_) at 30–40 mmHg. Anesthesia was maintained with propofol 0.1–0.2 mg/kg/min and remifentanil 0.2–0.3 µg/kg/min to maintain a BIS of 40–60. Fluid and vasoactive drugs were administered to maintained intraoperative haemodynamics within the appropriate range during surgery by experienced anesthesiologists. Following surgery, 0.15 µg/kg sufentanil and parecoxib 40 mg IV were used for postoperative analgesia. A patient-controlled intravenous analgesia pump with sufentanil was administered to all patients after being transferred to the recovery room.

### Cognitive function and pain measurement

Two neuropsychological tests were performed before and 7 days after surgery. Cognitive function was tested in a quiet environment by an experienced psychiatrist using the MMSE and Montreal Cognitive Assessment (MoCA). The MMSE [2] is the most widely used cognitive screening test and a well-accepted measure in clinical practice. It tests orientation, attention, calculation, and memory registration, as well as recall, language, and visuospatial skills. The MoCA [10] is a proven and useful cognitive screening tool with high sensitivity and the ability to assess many cognitive domains. It includes scores for memory, visuospatial ability, and executive functions, as well as attention, language, abstraction, naming, and orientation. The MoCA Beijing version was standardized for Chinese. Both tools can be performed in a short time of 5–15 min. In this study, cognitive decline in any test was defined as a decline in score by ≥ 1 SD from the preoperative subjects (*n* = 70) for that test. Patients were considered to exhibit POCD with cognitive decline in both tests, as mentioned in previous studies [11].

Median arterial pressure (MAP) and HR were monitored before anesthesia induction (t0), 30 min after surgery (t1), and after extubation (t2). The following variables including remifentanil dosage, operation time, recovery time, sufentanil consumption during the first 24 h postoperatively, and the incidence of adverse effects, including postoperative nausea/vomiting, postoperative hypotension, pruritus, and respiratory depression (RR < 10, SPO2 < 94 % during O_2_ inhalation) were also recorded. In addition, the visual analog scale (VAS) (0, no pain, 10 unbearable severe pain) was used to assess the degree of pain at rest 6 h (T1), 24 h (T2), and 48 h (T3) after surgery.

### Assay of plasma samples

Venous blood samples (5 mL) were collected from all patients 1 day before surgery (baseline), and 1 (day 1) and 3 days (day 3) after surgery, respectively. Blood samples were centrifuged at 1000 × g for 20 min at 4 °C, and the plasma was separated and stored at − 80 °C for future analysis. The concentrations of HMGB1, TNF-α, and IL-6 in the plasma were measured using a standard enzyme-linked immunosorbent assay method with commercially available kits.

All data were collected by the same physician who was unaware of the patients’ details and grouping but familiar with the assessment methods and indices used in the study.

### Statistical analysis

The sample size was calculated using IBM SPSS Sample Power version 3.0 (IBM Corp., Armonk, New York, USA). The study was powered using a pilot study to detect the incidence of POCD 7 days after surgery between the Q and C groups. A pilot study that included 20 patients showed a POCD rate of 5.7 % in the Q group and 33.8 % in the C group. As a result, at least 28 patients were required in each group to achieve a power of 0.8 with 0.05 alpha. Therefore, we planned to enroll 35 patients for each group to account for the possibility of attrition.

Statistical analyses were conducted using the Statistical Package for the Social Sciences software version 24.0 (IBM Corp., Armonk, New York, USA).

Normality was tested using the Kolmogorov-Smirnov test. Descriptive statistics are expressed as the mean ± SD, number of cases (n), and the percentage (%), median, and interquartile range. Intragroup comparisons were performed using the Student’s t-test or Mann-Whitney U test. Qualitative variables were compared using the chi-square (χ2) test or Fisher’s exact test. A repeated-measures analysis of variance was applied for comparisons between the two groups at different time points. A *P*-value of < 0.05 was considered statistically significant.

## Results

A total of 70 patients were enrolled in the study. Six patients were excluded because they met the exclusion criteria (*n* = 2) and declined consent (*n* = 4). Sixty-four patients were randomly divided into two groups using a random number generator: the QLB group (Q group) and control group (C group). Two patients were excluded from the Q group due to an unsuccessful quadratus lumborum block (*n* = 1) and being unexpectedly admitted to the intensive care unit (ICU; *n* = 1). Three patients were excluded from the C group due to unexpected ICU admission (*n* = 3). Thirty patients in the Q group and 29 patients in the C group ultimately completed the trial (Fig. 1). The baseline characteristics including age, sex composition, body mass index, ASA classification, comorbidity, and operative characteristics, are presented in Table 1.

**Table 1:** Baseline characteristics of the patients

		Q group (*n*=30)	C group (*n*=29)	*P*
Age (years)		71.2±5.3	71.2±5.2	0.467
Sex ratio [case(%)]	male	22 (73.3%)	19 (65.5%)	0.514
	female	8 (26.7%)	10 (34.5%)	
BMI (kg/m^2^)		21.6±3.2	22.1±3.3	0.527
ASA [case(%)]	I	7 (24.1%)	8 (26.7%)	0.697
	II	20 (69.0%)	17 (56.7%)	
	III	2 (6.9%)	5 (16.6%)	
Education (years)		7.2±2.0	7.1±2.2	0.910
Operative time (min)		203.0±58.3	205.7±55.3	0.856

Data are presented as the mean ± SD or number (%). Continuous data were compared by Student's t-test; categorical data were compared by Fisher's exact test; ranked data were compared by Mann-Whitney U test

*BMI* Body mass index, *ASA* American Society of Anesthesiologists

### Cognitive function score and incidence of POCD

**Fig. 3:**
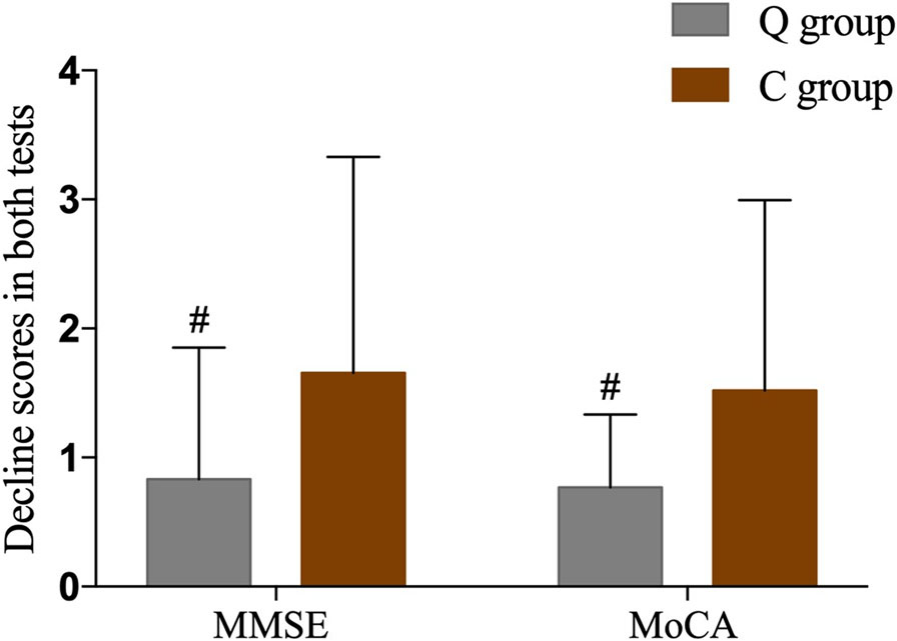
The decline scores in MMSE and MoCA tests between the two groups, Error bars represent the mean ± SD, compared with group C, ^#^*P* < 0.05. Data were compared by Student’s t-test. MMSE = Mini-Mental State Examination, MoCA = Montreal Cognitive Assessment

Patients showed no significant difference in the MMSE and MoCA scores between the two groups 1 day before surgery (*P* **>** 0.05). Cognitive scores of MMSE and MoCA in the C group 7 days after surgery were decreased compared with the preoperative scores (*P* < 0.05), while there was no difference in the Q group (*P* **>** 0.05; Table 2). Compared with C group, the decline scores in both MMSE and MoCA tests in Q group were significantly reduced between 1 day before surgery and 7 days after surgery (*P* < 0.05; Fig. 3).

**Table 2:** The MMSE and MoCA scores of the patients

	Group	N	1 day before surgery	7 days after surgery
MMSE	Q	30	27.5±1.78	26.8±1.30^#^
	C	29	27.7±1.82	26.0±1.60^*^
MoCA	Q	30	28.5±1.80	27.8±1.68^#^
	C	29	28.3±1.85	26.8±1.93^*^

Compared with group C, ^#^*P*<0.05, compared with 1 day before surgery, ^*^P<0.05. Data were compared by repeated-measures analysis of variance

*MMSE* Mini-Mental State Examination, *MoCA* Montreal Cognitive Assessment

Two patients (6.7 %) developed POCD in the Q group and eight (27.6 %) in the C group at 7 days after surgery, with statistically significant differences (*P* = 0.032, *x2 = 4.584*).

### Perioperative hemodynamic, anesthetic variables, and VAS scores

A repeated-measures ANOVA was applied for comparisons MAP and HR between the two groups. The hypothesis of Sphericity was not satisfied (*P* < 0.05), and the Epsilon correction coefficient was used for correction. The interaction between group and time had statistically significant effects on MAP and HR (*P* < 0.05). Through post hoc tests, there were no significant differences in the MAP and HR between the two groups at t0 and t2. The MAP and HR of group C at t1 were significantly higher than at t0 (*P* < 0.05). MAP in the Q group at t1 was significantly lower than that in the C group (*P* < 0.05; Table 3).

**Table 3:** Intraoperative MAP and HR of the patients

	Group	N	t0	t1	t2
MAP	Q	30	96.8±11.3	97.6±13.3^#^	96.4±10.9
	C	29	97.8±12.2	105.8±11.6^*^	99.3±11.5
HR	Q	30	69.1±9.9	72.2±12.9	66.4±11.0
	C	29	67.6±10.7	76.3±15.1^*^	68.9±12.5

Compared with group C, ^#^*P*<0.05, compared with t0, ^*^P<0.05. Data were compared by repeated-measures analysis of variance

*HR* heart rate, *MAP* mean artery pressure

**Fig. 4:**
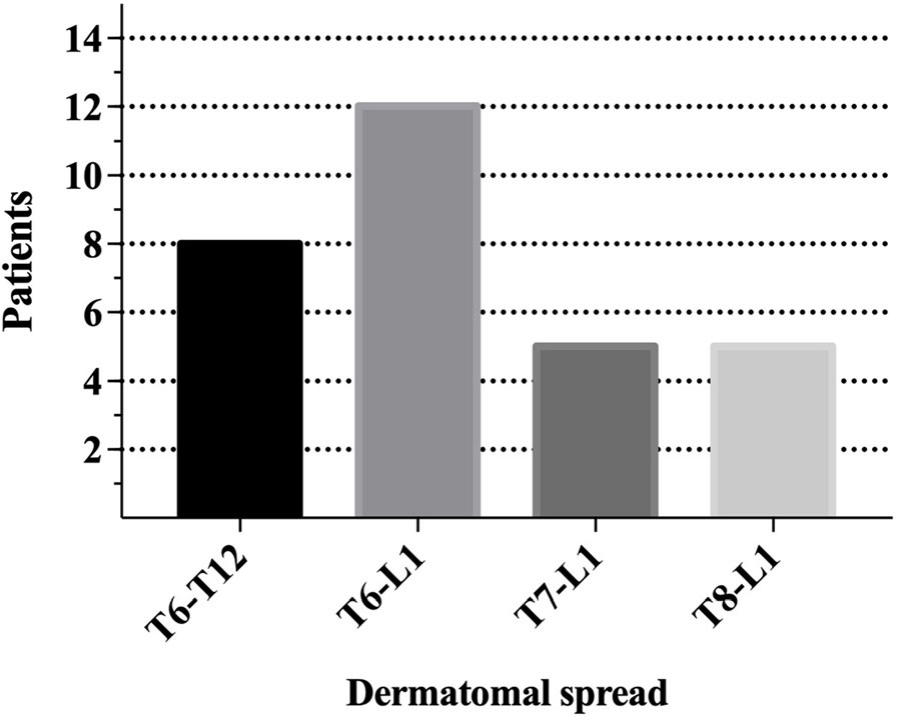
Dermatomal spread in patients who received QLB

The remifentanil dosage during surgery and sufentanil consumption during the first 24 h postoperatively in the Q group were significantly lower than those in the C group (*P* < 0.05). The recovery time was shorter in the Q group than in the C group (*P* < 0.05; Table 4). Fifteen minutes after performing QLB, the dermatomes of the sensory block in patients of group Q reached L1 in caudal and T6 in cranial (Fig. 4).

**Table 4:** Anesthetic variables and adverse effects

	Q group (*n*=30)	C group (*n*=29)	*P*
Remifentanil dosage (ug)	1.26±0.4^#^	1.53±0.4	0.013
Sufentanil consumption (ug)	35.77±10.2^#^	41.72±11.0	0.036
Time of recovery (min)	18.3±6.3^#^	22.1±6.6	0.028
PONV	9 (3.0%)	11 (3.8%)	0.520
Postoperative hypotension	0	0	
Pruritus	8 (26.7%)	6 (20.0%)	0.590
Respiratory depression	0	0	

Compared with group C, ^#^*P*<0.05. Continuous data were compared by Student's t-test; categorical data were compared by Fisher's exact test

*PONV* postoperative nausea and vomiting

Compared with the C group, the VAS at rest was significantly lower in the Q group at all times after surgery (*P* < 0.05; Table 5).

**Table 5:** Visual analog scores for pain at rest in each group (score, M[IQM])

	Q group (*n*=30)	C group (*n*=29)	*P*-value
VAS at rest (0-10)			
T1	3(2-4) ^#^	4(3-5)	0.006
T2	2(1-3) ^#^	3(2-3)	0.011
T3	1(1-10) ^#^	1(1-2)	0.030

Compared with group C, ^#^*P*<0.05. Data were compared by Mann-Whitney U test

*VAS* visual analog scores

### Serum levels of HMGB1, TNF-α, and IL-6

**Fig. 5:**
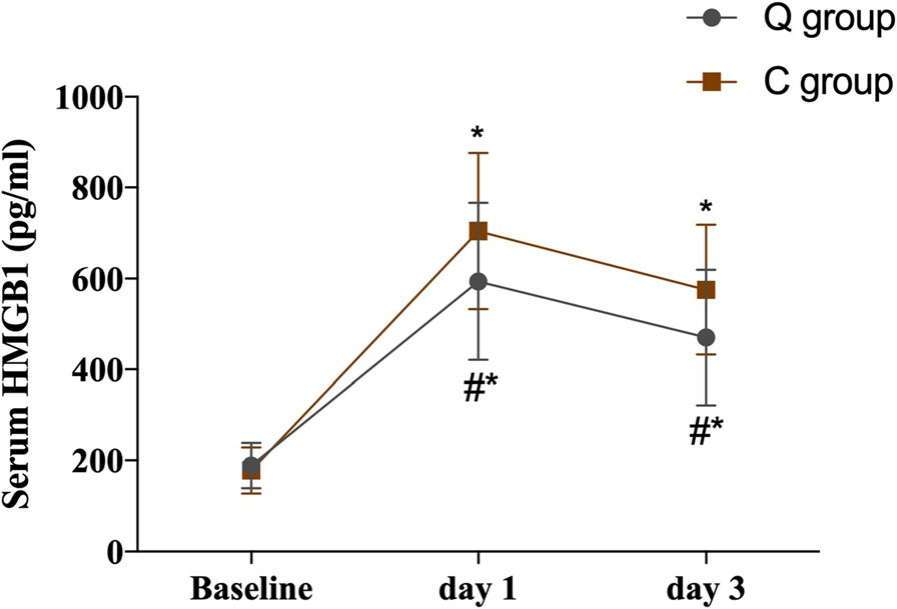
Serum levels of HMGB1 at different time points between the two groups. Error bars represent the mean ± SD, compared with group C, ^#^*P* < 0.05, compared with baseline, ^*^P < 0.05. Data were compared by repeated-measures analysis of variance. HMGB1 = high mobility group box 1

**Fig. 6:**
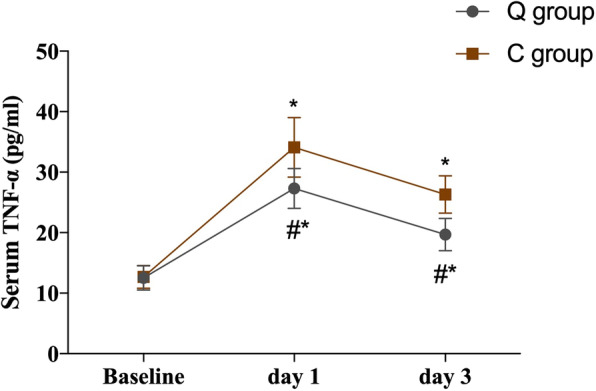
Serum levels of TNF-α at different time points between the two groups. Error bars represent the mean ± SD, compared with group C, ^#^*P* < 0.05, compared with baseline, ^*^P < 0.05. Data were compared by repeated-measures analysis of variance. TNF-α = tumor necrosis factor-α

**Fig. 7:**
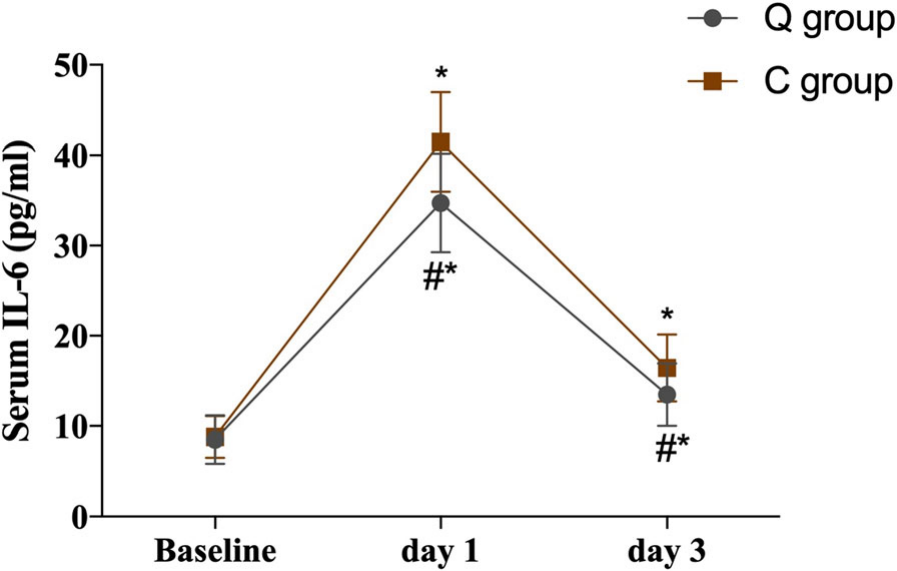
Serum levels of IL-6 at different time points between the two groups. Error bars represent the mean ± SD, compared with group C, ^#^*P* < 0.05, compared with baseline, ^*^P < 0.05. Data were compared by repeated-measures analysis of variance. IL-6 = interleukin-6

A repeated-measures ANOVA was applied for comparisons serum HMGB1, TNF-α, and IL-6 between the two groups. The hypothesis of sphericity was not satisfied (*P* < 0.05), and the Epsilon correction coefficient was used for correction. The interaction between group and time had statistically significant effects (*P* < 0.05). Through post hoc tests, the serum levels of HMGB1, TNF-α, and IL-6 were significantly higher at 1 and 3 days after surgery than at baseline in both the Q and C groups (*P* < 0.05). The peak levels were reached on day 1 and declined gradually on day 3 after surgery. Compared with the C group, the serum levels of HMGB1, TNF-α, and IL-6 were significantly lower in the Q group at days 1 and 3 postoperatively (*P* < 0.05; Table 6; Figs. 5, 6 and 7).

**Table 6:** Serum HMGB1, TNF-α, and IL-6 at different time points (pg/ml)

Group	Time point	HMGB1	TNF-α	IL-6
Q	Baseline	188.6±50.2	12.5±2.0	8.53±2.67
	Day 1	594.1±172.9^*#^	27.3±3.3^*#^	34.72±5.46^*#^
	Day 3	470.7±149.2^*#^	19.7±2.7^*#^	16.44±3.72^*#^
C	Baseline	178.1±51.2	12.7±1.9	8.80±2.32
	Day 1	704.4±171.8^*^	34.1±4.9^*^	41.47±5.52^*^
	Day 3	575.2±142.4^*^	26.3±3.1^*^	13.49±3.49^*^

Compared with group C, ^#^*P*<0.05, compared with baseline, ^*^P<0.05. Data were compared by repeated-measures analysis of variance

*HMGB1* high mobility group box 1, *TNF-α* tumor necrosis factor-α, IL-6=interleukin-6

## Discussion

The average life expectancy in China has increased from 74.83 years in 2010 to 77.3 years in 2019. With the development of modern medicine, an increasing number of elderly patients receive surgical treatment. Neurological complications, mainly manifested as cognitive impairment, have gradually become a common postoperative complication [12]. Cognitive change is generally observed in patients over a period after surgery. The incidence of POCD in patients (> 60 years) undergoing major noncardiac surgeries was 25.8 % 7 days after surgery and 9.9 % three months after surgery [13].

In our study, 27.6 % of patients in the control group experienced POCD compared with 6.7 % of patients in the Q group (6.7 %) 7 days after surgery. The incidence of POCD in the control group was similar to the results of a study in patients after elective surgery [11] but slightly different from the findings confirmed by Monk et al. [13]. This may be attributed to different cognitive tests and different types of surgery being used in the studies.

Previous investigations have identified several risk factors associated with cognitive dysfunction, including age, education years, large surgical trauma, anesthesia process, and acute postoperative pain [14]. It has been proven that surgical stress and acute postoperative pain are closely related to POCD. Wennberg et al. [15] showed that patients who received inadequate analgesia after a hip fracture might lead to impaired cognitive status. However, proper anesthetic management and effective postoperative analgesia can reduce the incidence of POCD [16]. In our previous study, it was confirmed that QLB was an effective analgesic method for abdominal surgery with few adverse effects [17]. QLB can alleviate postoperative pain, relieve perioperative stress response, and promote patients’ rehabilitation [18].

The present study showed that the fluctuations of intraoperative MAP and HR in Q group were less pronounced than that in C group, and the intraoperative remifentanil dosage and postoperative sufentanil dosage of Q group were significantly lower. The VAS was significantly lower at all time points after the operation, and the recovery time was shorter in the Q group. These results suggest that QLB can maintain intraoperative hemodynamic stability, reduce the consumption of opioids, and provide effective analgesia.

The current study revealed that QLB for postoperative analgesia was associated with higher MMSE scores, higher MoCA scores, and a lower incidence of POCD 7 days after surgery in elderly patients undergoing laparoscopic radical gastrectomy. Our results indicate that QLB analgesia can improve postoperative cognitive function in elderly patients, which may be attributed to the effective postoperative pain relief of QLB and low perioperative opioid consumption. It has been reported that the use of opioids may influence older patients’ cognitive function [19].

A neuroinflammatory cascade is the leading mechanism of POCD. Inflammatory responses, including the release of TNF-α and IL-6 induced by surgical trauma, can directly affect the central nervous system by crossing the compromised blood-brain barrier [20]. Previous studies have shown that TNF-α and IL-6 levels increased postoperatively and were closely related to cognitive decline [21]. Animal studies have directly implicated that proinflammatory cytokines and molecular mechanisms such as HMGB1, which are involved in inflammation induced by surgery, can cause neuroinflammation and subsequently lead to neurocognitive decline [16]. As a nuclear protein, HMGB1 plays an important role in the inflammatory process, mediates hippocampal inflammation, and causes cognitive decline [22]. Lin and colleagues found that increased levels of HMGB1 after gastrointestinal surgery appear to be related to POCD [5]. This study demonstrated that serum HMGB1, TNF-α, and IL-6 levels in the Q group were significantly lower than those in the C group 1 and 3 days after surgery, indicating that QLB analgesia combined with general anesthesia for laparoscopic radical gastrectomy can significantly inhibit the systemic inflammatory response and subsequently alleviate cognitive decline.

As a regional block for postoperative analgesia, QLB blocks only the abdominal somatosensory nerve with few adverse effects. It can provide effective analgesia after surgery and reduce the harmful surgical, traumatic stress, and inflammatory response to maintain homeostasis of the internal environment. Therefore, it can reduce the secretion of HMGB1, TNF-α, and IL-6, which are important in improving cognitive dysfunction.

There were also several limitations to this study. First, it was conducted in a single center with a small sample size. Furthermore, the follow-up time was as short as 7 days. Therefore, multicenter trials with large sample sizes and a longer follow-up period are required to confirm the findings of the current study. Second, we only used two common cognitive tests in our study, and multiple tests with different focus areas should be performed in further studies. Third, based on previous research, we used 40 mL of 0.3 % ropivacaine for peripheral nerve block, and none of the patients had toxic reactions. However, it is necessary to verify the optimal local anesthetic concentration and volume of QLB in elderly patients.

## Conclusions

QLB combined with general anesthesia could improve postoperative cognitive function in elderly patients undergoing laparoscopic radical gastrectomy, which may be associated with inhibition of release of HMGB1, TNF-α, and IL-6.

## Data Availability

The datasets used during the current study are available from the corresponding author on reasonable request.

## Abbreviations

ASA: America Society of Anesthesiologist
BIS: Bispectral index
BMI: Body mass index
ECG: Electrocardiogram
HMGB1: High mobility group box protein 1
HR: Heart rate
ICU: Intensive care unit
IL-6: Interleukin-6
IQM: Interquartile-range
MAP: Mean arterial pressure
MMSE: Mini-Mental State Examination
MoCA: Montreal Cognitive Assessment
MAP: Median arterial pressure
PetCO_2_: End-tidal carbon dioxide pressure
POCD: Postoperative cognitive dysfunction
PONV: Postoperative nausea and vomiting
QLB: Quadratus lumborum block
RR: Respiratory rate
SpO_2_: Percutaneous oxygen saturation
TNF-α: Tumor necrosis factor-α
VAS: Visual analogue scale
